# The Beneficial Effect of Proanthocyanidins and Icariin on Biochemical Markers of Bone Turnover in Rats

**DOI:** 10.3390/ijms19092746

**Published:** 2018-09-13

**Authors:** Nada Oršolić, Johann Nemrava, Željko Jeleč, Marina Kukolj, Dyana Odeh, Svjetlana Terzić, Rajko Fureš, Tomica Bagatin, Dinko Bagatin

**Affiliations:** 1 Division of Animal Physiology, Faculty of Science, University of Zagreb, Rooseveltov trg 6, 10000 Zagreb, Croatia; kukoljmarina@gmail.com (M.K.); dyana.odeh@biol.pmf.hr (D.O.); 2 Polyclinic Bagatin, Grada Vukovara 269a/10, 10000 Zagreb, Croatia; johann.nemrava@gmail.com (J.N.); tomica.bagatin@poliklinikabagatin.hr (T.B.); dinko.bagatin@poliklinikabagatin.hr (D.B.); 3 Department of Orthopaedic Surgery, Specialty Hospital St. Catherine, 49210 Zabok, Croatia; zjelec@yahoo.co.uk; 4 Croatian Veterinary Institute, Savska cesta 143, 10000 Zagreb, Croatia; terzic@veinst.hr; 5 Department of Gynaecology and Obstetrics, General Hospital Zabok, 49210 Zabok, Croatia; rajko.fures@bolnica-zabok.hr

**Keywords:** proanthocyanidins, icariin, retinoic acid-induced bone loss, rats, bone markers

## Abstract

Nutrition is an important factor that influences bone metabolism, the endocrine and/or paracrine system, and bone-active mineral elements homeostasis. We studied antiosteoporotic effects of grape seed proanthocyanidins extract, icariin or alendronate (ALN) in retinoic acid-induced (13cRA) bone loss in rats. Proanthocyanidins and icariin have beneficial effects on bone health; they have improved the bone weight reduction, the length and the diameter of the bone, calcium, and phosphorus content in bone ash, bone mineral density (BMD), the biochemical markers of bone turnover and uterus atrophy induced by 13cRA. All results suggest that proanthocyanidins and icariin reverse osteoporosis in 13cRA rats by stimulating bone formation or regulating bone resorption by their antioxidative and estrogenic-like activity without toxic side-effects observed in ALN treatment.

## 1. Introduction

Higher intake of fruits and vegetables has been associated with improved bone mineral density (BMD) or bone mineral content (BMC) and bone health [1]. Most of the studies have focused primarily on the isoflavone subclass of flavonoids, mainly contained in soy rich foods. Other classes of flavonoids, including proanthocyanidins or icariin, are also abundant in plants and contribute significantly to daily flavonoid intake. Proanthocyanidins, better known as condensed tannins, are formed by polymerization of a basic flavan-3-ol unit and they are potent antioxidants. In the human diet, they are found in aronia, such as fruits, apples, pears, grapes, as well as in cocoa beans, wine and tea. Icariin is a flavonol, a class of flavonoid, and it has been identified as the major active ingredient in *Herba epimedii* and it can be derived from several species of plants in the *Epimedium* family.

Macro- and micro-phytonutrients contribute to the skeletal health by supporting bone matrix production and mineralization [2]. Beneficial effects on bones have been attributed to several potential factors, including acid-base balance, potassium, fiber, calcium, magnesium contents or other micronutrients such as vitamin K. Dietary factors independent of calcium and/or vitamin D may influence bone and mineral homeostasis and may be important for long-term bone health [3,4]. In addition, it is recognized that calcium and proteins, the most important nutrients for supporting the skeleton, are best obtained from the food sources and that consumption of flavonoids may improve calcium absorption in small intestine and may enhance activity of vitamin D receptor (VDR), responsible for regulation of the gene expression related to calcium homeostasis [5]. According to Diaz de Barboza et al. [6] intestinal Ca^2+^ absorption depends on the of GSH content and GSH deficiency can inhibit the intestinal Ca^2+^ absorption by modifying the pathways and molecules involved in its transfer. The lack of GSH, subsequent to drug-induced oxidative stress, leads to the process of apoptosis or autophagy that can be blocked by antioxidant, antiapoptotic, and anti-autophagic effect of natural antioxidants [7].

Bioactive compounds, including proanthocyanidins and icariin, in some food show anti-resorptive and anti-inflammatory properties, high antioxidant capacity and phytoestrogenic activity [8,9,10]. Phytoestrogens can attenuate bone loss associated with oestrogen deficiency by acting similarly to oestrogen, as well as through antioxidative properties and their influence on antioxidant enzyme activity in both animal and human studies [11]. Similar to oestrogen involved in growth, differentiation and function of reproductive and other tissues including the bone [12], phytoestrogens may inhibit the differentiation of osteoclasts and stimulate osteoblastic bone formation.

Despite the fact that hormone replacement terapy improves bone mineral density, its use is limited due to several complications including the increase of risk in postmenopausal women for developing breast cancer, uterine bleeding, stroke, thrombosis and cardiovascular disease [13,14,15]. Earlier findings are suggestive of high need for discovering another form of osteoporosis treatment. Additional challenge in osteoporosis therapy is different incidence of osteonecrosis of the jaw which seems relatively low in patients receiving oral bisphosphonate for osteoporosis or Paget’s disease and considerably higher in patients with malignancy receiving high doses of intravenous bisphosphonate [16,17]. In addition, it increases the risk of developing cancer in post-menopausal women [13] and atypical femoral fractures as defined by the American Society for Bone and Mineral Research (ASBMR) after long-term bisphosphonate therapy. Thus, there is a need for safe and effective compounds that can inhibit bone loss.

Since proanthocyanidins and icariin are powerful antioxidants and phytoestrogens [8,9,10] they may be useful in the treatment of osteoporosis induced by retinoic acid. In present study we have investigated the therapeutic effect of proanthocyanidins and icariin on 13 cis-retinoic acid (13cRA) induced osteoporosis in rats as a drug-induced bone loss model.

Therapeutic effect of proanthocyanidins and icariin on the retinoic acid-induced osteoporosis was compared with the effect of alendronate, which is mostly used as an anti-osteoporotic drug.

## 2. Results

### 2.1. Body Weights

**Figure 1:**
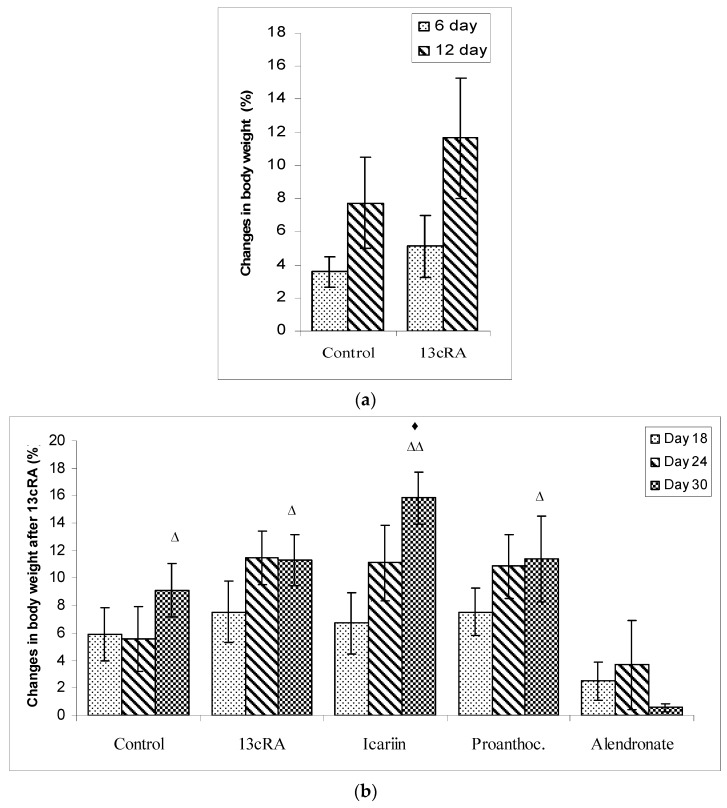
Changes in body weight at different time points: (**a**) during treatment with retinoic acid, (**b**) effect of icariin, proanthocyanidins and alendronate on the body weight change in rats after retinoic acid-induced bone loss. Rats were administered *ig* with retinoic acid suspension (80 mg kg^−1^) once daily for 14 days. After inducing osteoporosis with 13cRA, rats were administered *ig* with icariin (100 mg kg^−1^), proanthocyanidins (100 mg kg^−1^) or alendronate (40 mg kg^−1^) once daily for next 14 days. The animals were weighed just before the start of the experiment (starting weight of animals 225–250) and during the experiment on days 6, 12, 18, 24 and 30. The percentage change in weight was calculated for individual animals as Percentage change in weight = Final weight-Initial weight × 100/Final weight. Number of rats per group: 7. ^♦^ Statistically significantly different compared to control (^♦^ *p* < 0.05) on day 30. ^Δ^ Statistically significantly different compared to alendronate (^Δ^ *p* < 0.05; ^ΔΔ^ *p* < 0.01) on day 30.

Percentage of the animal body weight change during the experiment is shown in Figure 1. In all of the control animals and/or animals treated with a 13cRA weight gain was observed; percentage of body weight change of the control group on day 6 and on day 12 was 3.58% and 7.74% while in the animals treated with 13cRA percentage of body weight change was 5.10% and 11.66% respectively. Weight gain of rats treated with 13cRA showed an increase of 1.52–3.9% compared to the healthy control group (Figure 1a). After causing osteoporosis with 13cRA, treatment of animals with proanthocyanidins and icariin in a dose of 100 mg kg^−1^ led to a further weight gain in the period from 18th to 30th day for: icariin 6.73–15.86%; and proanthocyanidins 7.52–11.40% respectively. In the group treated with alendronate 40 mg kg^−1^ we have observed a decrease in body weight of 2.50% to 0.55% in the period from 18th to 30th day. In same period, the healthy control group and the group treated with 13cRA (model of osteoporosis) showed a further weight gain of 5.88–9.10% and 7.54∓11.28% (Figure 1b).

After two weeks of treating the rats with 13cRA, there was no significant change in the final body mass between the 13cRA and healthy controls. Statisticaly significant difference in body weight was observed between the control (*p* < 0.05), icariin (*p* < 0.01), and proanthocyanidins (*p* < 0.05) groups in relation to alendronate at dose of 40 mg kg^−1^ and between the control and icariin (*p* < 0.05) on day 30 (Figure 1b).

Treatment with 13cRA resulted in a significant increase in ALP (*p* < 0.05) compared to control (Table 1) and plasma total protein compared to icariin (*p* < 0.05). LDH level in 13cRA group increased in relation to control (725.66 ± 157.59 vs. 464.50 ± 130.97) but without statistical significance. The oral administration of proanthocyanidins and icariin via a gavage needle to osteoporotic rats caused a significant decrease in LDH levels (*p* < 0.01; *p* < 0.05) in relation to 13cRA while alendronate caused a significant decrease in ALP levels (*p* < 0.05) in relation to 13cRA. In the animals treated with icariin the value of total proteins was lower compared to 13cRA (*p* < 0.05) and proantociyanidins (*p* < 0.01) while other proteins, and blood metabolites were unchanged with respect to control and 13cRA (data not shown).

Results in Table 2 indicated that alendronate caused a significant increase in total leukocyte count (*p* < 0.05) in relation to control and platelet count compared to icariin (*p* < 0.05) (data not shown). Differential blood analysis shows increased monocyte and neutrophil counts (*p* < 0.05 and *p* < 0.05) in the alendronate treated group compared to the control (Table 2).

### 2.2. Effect of Icariin, Proanthocyanidins and Alendronate on Bone Health and Metabolism in Rats with Retinoic Acid-Induced Bone Loss

The relative weight (g/100 g) of the left femur (Table 3) was significantly higher in healthy control group (*p* < 0.05) and alendronate treated group (*p* < 0.05) compared to 13cRA. The relative weight of the right femur (Table 3) was significantly higher in healthy control group (*p* < 0.001), icariin treated group (*p* < 0.05) and in the alendronate treated group (*p* < 0.01) compared to 13cRA group.

The results of geometric bone parameters of the right femur are also translated to 100 g body weight. We measured the length of the bone (large trochanter-condyle), anterior-posterior length (AP) and medio-lateral length (ML) of the proximal part of the bone, the middle part of the bone (diaphysis) and distal bone (Table 4). The results show statistically significant increase in the length of the bone between icariin treated group and 13cRA model group (*p* < 0.001).

No significant differences were observed between groups for the ML and AP diameters of the proximal epiphysis. The ML diameter of the distal epiphysis was significantly lower in 13cRA model group compared with healthy control (*p* < 0.05) and AP diameter compared to icariin (*p* < 0.01).

**Figure 2:**
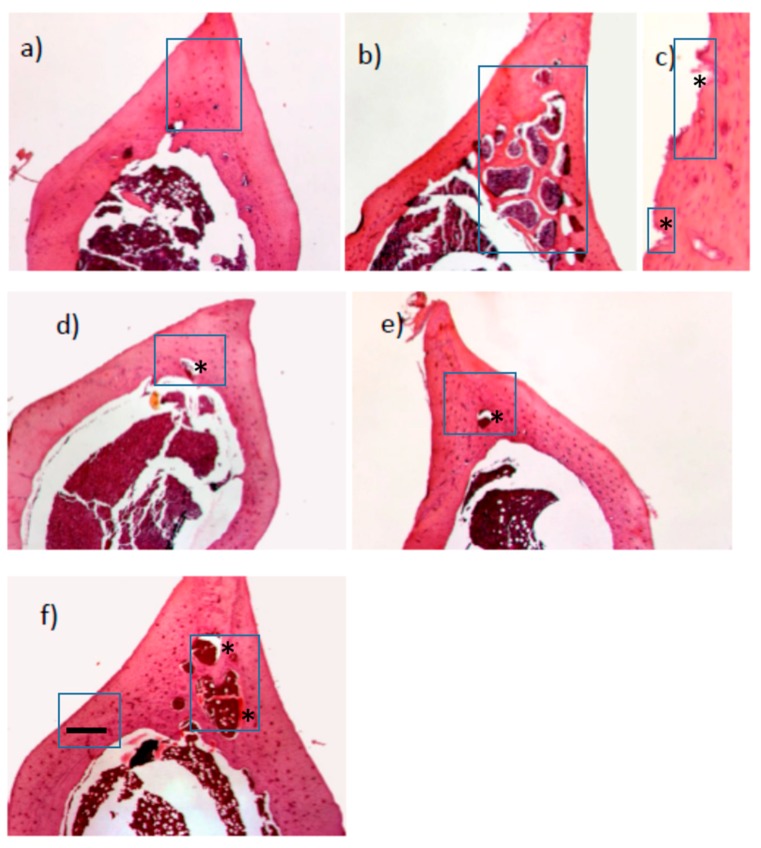
Histological examination of rats’ bone stained section with HE. (**a**) Control group showed normal architecture of the bone tissue (shown in box); (**b**) Retinoic acid-treated group (osteoporotic model) showed formation of multiple large intracortical cavities (shown in box) and thinning of the cortex and eroded endosteal bone surface (shown in box with *) (**c**); Proanthocyanidins (**d**) or icariin (**e**) showed complete recovery with essential features of the normal bone and complete formation of trabeculae and thickness with one or two small intracortical cavities (shown in box with *); (**f**) alendronate showed signs of improvemaent of femoral structure and the increased thickness (shown in box with ▬), size of the intracortical cavities are significantly reduced, while the inner surface of the cortex still shows irregularities in certain places (shown in box with *).

The bone architecture of the distal part of the femoral diaphysis was analysed after HE staining. The rats of control group revealed normal compactness of the diaphysis and competent trabeculae and periosteum and endosteum without any irregularities (Figure 2a). The femoral cortex of 13cRA treated groups showed sparse and thin trabeculae, as well as loss of connectivity (Figure 2b). It is characterized by significant porosity caused by the formation of numerous intracortical cavities of different sizes and the partially eroded endosteal bone surface (Figure 2c). The cavities are partially obstructed by osteoids on the cortical side and subperiosteal osteoclastic osteolysis in midshaft of femur was found, as well as eroded both endosteal and periosteal surface. Diaphyseal cortex of osteoporotic group treated with proanthocyanidins and icariin showed nearly complete recovery with essential features of the normal bone and complete formation of trabeculae, and a thickness nearly similar to that of control group (Figure 2d,e). One or two small intracortical cavities were found and endosteal surface was smooth with only a few slight irregularities. Cortical bone of osteoporotic rats treated with alendronate showed signs of improvement of femoral structure. The thickness of the cortical bone has increased; the number and size of the intracortical cavities was significantly reduced, while the inner surface of the cortex still showed irregularities in certain places (Figure 2f).

**Figure 3:**
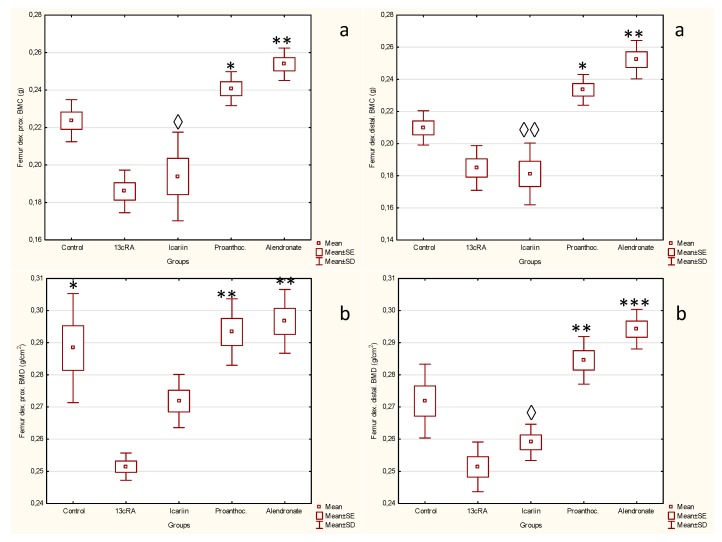
Effect of icariin, proanthocyanidins and alendronate on bone mineral content (**a**) bone mineral density (**b**) in rats with retinoic acid-induced bone loss. Rats were administered *ig* with retinoic acid suspension (80 mg kg^−1^) once daily for 14 days. After inducing osteoporosis with 13cRA, rats were administered *ig* with icariin (100 mg kg^−1^), proanthocyanidins (100 mg kg^−1^) or alendronate (40 mg kg^−1^) once daily for next 14 days. Bone mineral content (BMC) and bone mineral density (BMD) of each rat’s left femur were measured by dual-energy X-ray absorptiometry (DXA). BMD was calculated by the BMC of the measured area and reported as g/cm^3^. Number of rats per group: 7. (**a**) Statistically significant difference exists between: Prox., 13cRA vs. alendronate (** *p* ˂ 0.01), 13cRA vs. proanthocyanidins (* *p* < 0.05); icariin vs. alendronate (^◊^ *p* < 0.05);.Distal, 13cRA vs. alendronate (** *p* ˂ 0.01), 13cRA vs. proanthocyanidins (* *p* < 0.05); icariin vs. alendronate (^◊◊^ *p* < 0.01); (**b**) Statistically significant difference exists between: Prox., 13cRA vs. Control (* *p* < 0.05); 13cRA vs. proanthocyanidins (** *p* < 0.01); 13cRA vs. alendronate (^◊^ *p* < 0.01); Distal. 13cRA vs. proanthocyanidins (** *p* < 0.01); 13cRA vs. alendronate (*** *p* < 0.001); icariin vs. alendronate (^◊^ *p* < 0.05).

Bone mineral content and bone mineral density were measured in the proximal and distal metaphysis of the right femur (Figure 3). At day 30 femoral BMC in distal metaphysis of femur was significantly lower in the 13cRA group than proanthocyanidins or alendronate treated group (*p* < 0.05; *p* < 0.01). Differences in BMC at the proximal metaphysis of the femur between 13cRA and proanthocyanidins or alendronate treated groups were more marked than the differences in distal metaphysis part (*p* < 0.05; *p* < 0.001).

Data analysis revealed statistically significant increase in BMD in the proximal and distal metaphysis of the femur in osteoporotic treated group in comparation to osteoporotic model (13cRA) (Figure 3). Specifically, statistically significant difference in the proximal metaphysis of the bone exists between healthy controls and 13cRA (*p* < 0.05); in both proximal and distal metaphysis of the bone, between 13cRA and proanthocyanidins (*p* < 0.01) and between 13cRA and alendronate-treated group at dose of 40 mg kg^−1^ (prox.—*p* < 0.01; dist.—*p* < 0.001).

Figure 3 shows that treatment of animals with alendronate caused significant increase in the proximal and distal metaphysis of the femur in BMC and BMD compared to 13cRA and icariin.

The levels of calcium and phosphorus in the osteoporotic model (13cRA) group were significantly lower in relation to healthy control (*p* < 0.05 and *p* <0.05) and osteoporotic animals treated with and icariin (*p* < 0.05 and *p* < 0.05) or proanthocyanidins (*p* < 0.05; and *p* < 0.05). Content of calcium and phosphorus in the proanthocyanidins treated group increased by 21.65% and 19.30% compared to 13cRA. Icariin increased calcium level of 14.7%, and phosphorus level 16.65% in relation to 13cRA (Table 5).

No effects of the different treatment on the serum levels of calcium and phosphorus were found (Table 5).

The results in Table 5 showed no statisticaly significant difference between the groups for the serum vitamin D level.

### 2.3. Effect of Icariin, Proanthocyanidins and Alendronate on Osteoclast and Osteoblast Activities in Rats with Retinoic Acid-Induced Bone Loss

**Figure 4:**
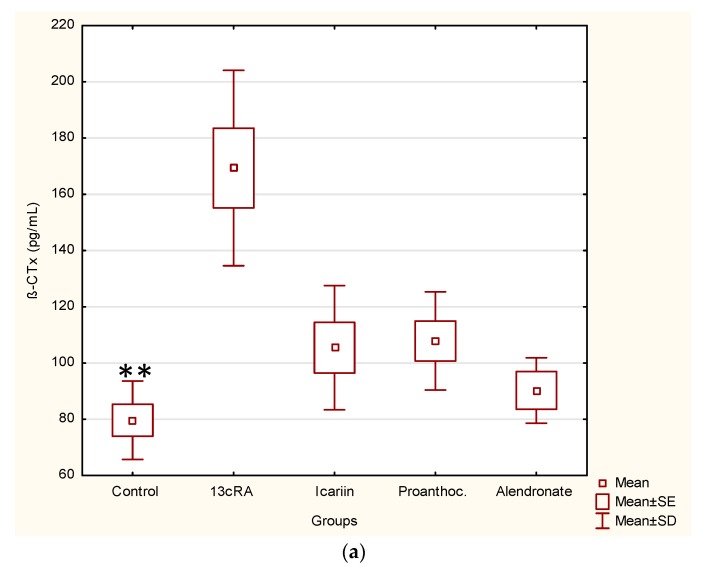

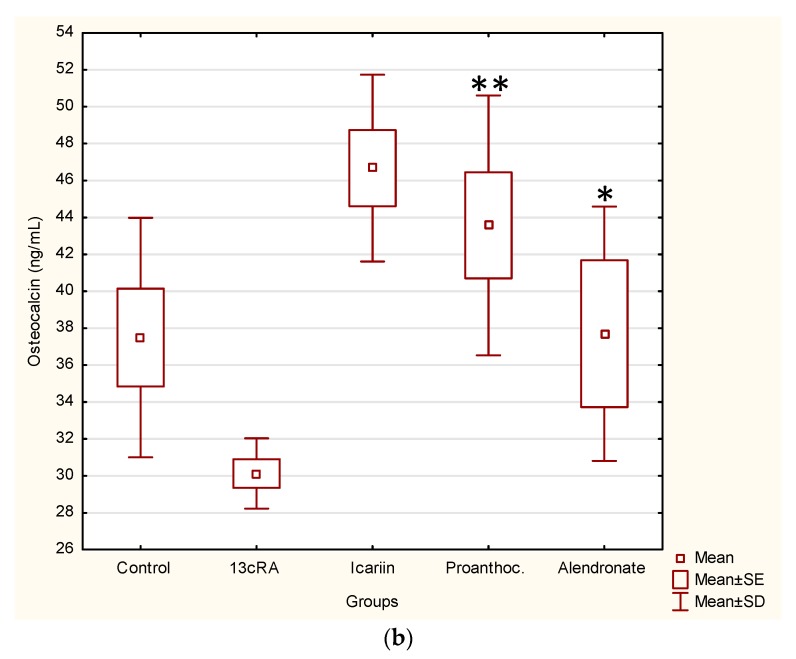
Effect of icariin, proanthocyanidins and alendronate on bone biochemical markers in rats with retinoic acid-induced bone loss. Bone biochemical markers (**a**) osteocalcin, a marker of bone formation and (**b**) β-CrossLaps (β-CTx) marker of bone resorption. Rats were administered *ig* with retinoic acid suspension (80 mg kg^−1^) once daily for 14 days. After inducing osteoporosis with 13cRA, rats were administered *ig* with icariin (100 mg kg^−1^), proanthocyanidins (100 mg kg^−1^) or alendronate (40 mg kg^−1^) once daily for next 14 days. Serum osteocalcin and β-CrossLaps (β-CTx) markers were measured by the **e**lectro**c**hemi**l**uminescence **i**mmuno**a**ssay “ECLIA”. Number of rats per group: 7. (**a**) Statistically significant difference exists between: 13cRA vs. control (** *p* < 0.01). (**b**) Statistically significant difference exists between: 13cRA vs. icariin (** *p* < 0.01); 13cRA vs. proanthocyanidins (* *p* < 0.05).

The intragastric administration of 13cRA at dose of 80 mg kg^−1^ body weight daily for 2 weeks resulted in a significant increase in the value of β-CTx (*p* < 0.01) while level of OC decreased by 7.35% in relation to the healthy control group (Figure 4). Data analysis revealed a statistically significant increase of serum osteocalcin in groups treated with icariin (*p* < 0.01) and proanthocyanidins (*p* < 0.05) (Figure 4) compared to 13cRA.

### 2.4. Effect of Icariin, Proanthocyanidins and Alendronate on Antioxidant and Anti-Inflammatory Markers in Rats with Retinoic Acid-Induced Bone Loss

Table 6 shows the effect of 13cRA on the redox status in kidney cells. Retinoic acid significantly increased the level of MDA in kidney in relation to control (*p* < 0.05) while the level of GSH, SOD and CAT activity was reduced (*p* < 0.05). Interestingly, alendronate shows increased levels of MDA, GSH (*p* < 0.01), SOD and CAT (*p* < 0.05; *p* < 0.01) activities compared to 13cRA while treatment with icariin or proanthocyanidins shows values similar to the healthy control group. CAT activity was statistically significant in icariin or proanthocyanidins treated groups in relation to 13cRA.

Liver MDA increased following 13cRA administration (*p* < 0.05) while GSH and SOD activity decreased but without statistical significance in relation to control group (Table 7). In relation to control, treatment with alendronate significantly increased the level of MDA in liver (*p* < 0.05). Intragastric administration of icariin increased GSH level and SOD activity in liver as compared to 13cRA (Table 7).

Serum cytokine analysis showed only significant changes in the 13cRA group where IL-1β, TNF-α and RANTES chemokines increased, while icariin or proanthocyanidins led to a visible decrease in these cytokines and RANTES chemokines with the values not being different from the healthy control group values. Alendronate treatment of rat showed increased level of IL-1β (data not shown).

### 2.5. Oestrogenic-Like Activity of Icariin, Proanthocyanidins and Alendronate in Rats with Retinoic Acid-Induced Bone Loss

Treatment with 13cRA caused uterine tissue atrophy (Table 8) in rats, showing that 13cRA induces osteoporosis partly through lack of oestrogen activity. In icariin treated group the relative uterus weight increased significantly compared to the 13cRA (*p* < 0.01), whereas the proanthocyanidins had no significant effect on the uterus (Table 8). Percentage of oestrogenic-like activity in relation to 13cRA is specified as follows: icariin increases relative uterine weight to the 43.42%, proanthocyanidins 20.91%, control 19.49% and alendronate 17.43% (Table 8).

## 3. Discussion

Many medications can cause secondary osteoporosis, including retinoic acid, glucocorticoids, and aromatase inhibitors in women, gonadotropin–releasing hormone agonists in men, as well as anticonvulsant medications, antiepileptics, proton pump inhibitors and other drugs in both sexes [18]. Hypervitaminosis A is known to cause accelerated bone metabolism and spontaneous fractures in laboratory animals and a high dietary intake has been associated with the development of osteoporosis [19].

According to our previous data and data from other researchers [20,21,22,23,24,25,26,27,28,29,30,31,32,33,34], retinoic acid is an ideal agent for the induction of acute osteoporotic model. 13cRA at a dose of 80 mg kg^−1^ during 14 days successfully causes osteoporotic changes in the rats through the mechanisms of increased oxidative stress (OS) and the formation of reactive radicals, reduced activity of osteoblasts and increased osteoclast activity, decreased oestrogen levels and the appearance of inflammatory cytokines which activate osteoclasts and cause osteoblasts apoptosis, reduction in vitamin D receptor activity, decreased absorption of Ca^2+^ in intestine, increase Ca^2+^ secretion by kidneys and the effects on the parathyroid hormone. In addition, hypervitaminosis A leads to the weakening and loss of bone through several mechanisms including direct effects of vitamin A on bone cells by suppressing the osteoblast activity and stimulating osteoclast formation [19,30] causing bone loss through reduction of radial bone growth [22], suppression of expression of PTH in vivo and its reduction in serum [23,31] and increased level of oxidative stress [24,31].

Based on the facts mentioned above this model of osteoporosis caused by 13cRA is the ideal model for studying antioxidative, anti-inflammatory, phytoestrogenic and regenerative capacity of proanthocyanidins and icariin on 13cRA-induced bone loss model.

In the present study, we found that 13cRA had an effect on bone marker parameters; a significant decrease of BMD value in the proximal (*p* < 0.05) part of the femur, Ca and P contents in the femur (*p* < 0.05, *p* < 0.05), as well as increase of βCTX and ALP (*p* < 0.01; *p* < 0.05) (Table 1 and Table 5, Figure 3 and Figure 4). Also, the geometrical and physical characteristics of the bone such as median weight, length and both diameters (anterior-posterior and medio-lateral) of femur in retinoic acid treated group were lower than in the control group but with no statistical difference except ML diameter of the distal epiphysis and AP diameter compared to icariin (*p* < 0.01) (Table 3 and Table 4). Our data also demonstrated that 13cRA caused uterine tissue atrophy in rats, and that it induces osteoporosis, which is based partly on lack of oestrogen (Table 8) as suggested by Liao and coworkers [31]. 13cRA showed no significant change in the final body mass between the 13cRA and healthy controls.

We found that 13cRA leads to an increase in MDA levels and decrease in GSH, SOD and CAT activity in liver and kidney of rats treated with 13cRA. Decrease in the activity of the antioxidant enzymes could likely lead to cytotoxic death due to accumulation of H_2_O_2_ resulting in oxidative damage and uterine tissue atrophy. Our study is also consistent with the findings [33] that demonstrated that retinoic acid reduces the level of oestrogens in blood. According to Almeida et al. [21] reactive oxygen species (ROS) are involved in oestrogen deficiency induced bone loss through increased osteoclast activity leading to an imbalance between the formation and resorption of bone [1,21,22,30,35]. It seems that oestrogen insufficiency generally results in changes of tissue metabolism and has resulted in the alternations of antioxidative/oxidative balance in liver and kidney. Thus, the generation of ROS appears as an early event that precedes intracellular GSH depletion and cell damage in retinoic acid hepatotoxicity. Retinoic acid-induced hepatocellular damage is marked by increased plasma AST, ALP; GGT, amylase, and LDH activities (Table 1). These features might be attributed to the metabolic activation of retinoic acid, which is considered a major mechanism of its toxicity.

However, our finding demonstrated that two weeks of treatment with proanthocyanidins, icariin or alendronate significantly decreased the BMC and BMD loss in the femur and inhibited the bone turnover markers in serum such as ALP, OC, and βCTx levels compared to the 13cRA group. In the present study, different responses were seen in Ca and P content in femur between rats that received proanthocyanidins, icariin or alendronate in relation to 13cRA. The geometrical and physical characteristics of the bone are increased in proanthocyanidins, icariin or alendronate-treated group. Icariin significantly improves the geometric characteristics of the femur and weight of the uterus. These results suggested that icariin exerts oestrogen-like activity; it had a similar effect as oestrogen on bone loss and uterine weight. Uterine weight in icariin-treated group was higher by 43.42% compared to 13cRA, while proanthocyanidins-treated group shows an increase of 20.91% compared to 13cRA. The activity of proanthocyanidins and icariin has been attributed to antioxidants present in its flavan-3-ol or catechin that show free radical scavenging activity [36,37]. According to our data, proanthocyanidins or icariin are an effective antioxidant and free radical scavenger with estrogenic and osteoblastic activity. It can potentially protect against age-associated bone loss or bone loss induced by drugs that are known to act by generation of ROS such as retinoic acid. The antioxidant activity of proanthocyanidins and icariin counteract the toxic effects of 13cRA and contributes to decreased activity of blood enzymes such as AST, ALP, amylase and LDH, which can be a good indicator of liver, kidney and other organs damage. In addition, it seems that proanthocyanidins or icariin positively affect calcium and phosphorus balance (Table 5), an essential mineral in bone metabolism; the increase of Ca and P absorption in intestine resulted in corresponding increase in bone mineral. These data are confirmed by other authors who demonstrated that non-digestible oligosaccharides of proanthocyanidins or icariin, acting as phytoestrogens, have osteogenic and protective characteristics in the rat model through stimulation of osteoblast activity mediated by oestrogen receptors or by increasing the production of other osteoblastic factors including insulin 1 like growth factor-1 (IG-F) [38,39].

It is known that oestrogen plays an important role in Ca^2+^ homoeostasis, and oestrogen deficiency results in a negative Ca^2+^ balance and bone loss (Table 5) in rats and in postmenopausal women [40]. In addition, the positive effect of proanthocyanidins and icariin could be based on the inhibition of production of proinflammatory cytokines, including TNF-α and IL-1β that are responsible for inducing Synoviolin expression, an E3 ubiquitin ligase, which directly destroy bone and cartilage through the production of metalloproteinase and the receptor of nuclear factor-kappa B (NF-κB) ligand that increase osteoclast activity for bone destruction [41,42,43].

Alendronate showed significant improvement in bone histology in alendronate-treated 13cRA-induced osteoporotic rats [1], ALP activity, BMD, Ca and P levels, bone markers and geometric characteristic of femur, but our findings suggest that alendronate is a local irritant [35] that increases the levels of MDA in liver and kidney as well as SOD and CAT activity in the osteoporotic kidney. The increase in MDA concentration, despite increased SOD and CAT activity in kidney, could have been due to the overproduction of ROS that exceeded the capacity of these antioxidant enzymes. The increase in GSH level may be a compensatory response by local cells attempting to protect kidney from the damaging effects of lipid peroxidation by increasing the levels of endogenous GSH.

Despite the positive effects of alendronate on bone after 13cRA-induced osteoporosis, several authors suggest that bisphosphonates often cause renal insufficiency and gastrointestinal adverse effects [44,45,46], which can be detected in the reduction of body weight (Figure 1b). According to Conwell and Chang [45], Perazzella and Markowitz [46], Sener et al. [47] and Shikama et al. [48] high doses of alendronate may cause inflammation through neutrophil and macrophage infiltration and oxidative damage in tissues as demonstrated in our results (Table 2 and Table 6). In addition, study from Shikama et al. [48] demonstrated that macrophages are involved in the inflammatory side effects of alendronate in mice and in RAW 264 cells in vitro by production IL-1β. The latest research shows that the application of bisphosphonates over a period longer than 5 years may increase the risk of atypical femur fractures [13,49]. According to this statement, proanthocyanidins or icariin as anti-inflammatory component could be a good substitute for alendronate, which induces a strong inflammatory effect [42,46,47,48], which may have been a contributing factor for several diseases [13].

In conclusion, our findings suggest that proanthocyanidins and icariin demonstrate a significant antioxidant and antiosteoporotic effect in retinoic acid induced osteoporotic rats by maintaining calcium and phosphorus homeostasis and increasing antioxidative enzymes that increase bone markers formation and bone mineral density while reducing induced lipid peroxidation. Alendronate is effective in treating osteoporosis induced by retinoic acid; positively affects all bone parameters but causes increased oxidative stress especially in the kidneys and causes a reduction in body weight. In addition, icariin exhibits a higher oestrogen-like activity than proanthocyanidins or alendronate. Furthermore, this study contributes to a better understanding of the relationship between flavonoids rich diet and health and based on our current scientific undestunding, proanthocyanidins and icariin offer great hope for the treatment secondary osteoporosis induced by retinoic acid.

## 4. Materials and Methods

### 4.1. Reagents

Isotretinoin (13cRA; 13 cis-Retinoic acid) (Accutane^®^, Hoffmann-La Roche Ltd., Basel, Switzerland), Alendronate (Alendor^®^70, Pliva, Zagreb, Croatia), Proanthocyanidins (Tianjin Jianfeng Natural Products R&D, Co. Ltd., Tianjin, China ) and Icariin, (C_33_H_40_O_15_; molecular weight: 676.67 g mol^−1^) were purchased from the National Institute for the Control of Pharmaceuticals and Biological Products (Beijing, China).

### 4.2. Proanthocyanidins and Icariin

Purified grape seed proanthocyanidin preparation was composed of 96.64% proanthocyanidins, which contained 6.1% catechin, 6.78% epicatechin, 55.59% dimeric forms, 11.91% trimeric forms, 6.55% tetrameric forms and small amounts of other polymeric forms [50]. Icariin is a chemical compound classified as a flavonol glycoside, a type of flavonoid. Before use, proanthocyanidin or icariin were dissolved in water. Proanthocyanidin or icariin were given to rats by intragastric application (*ig*) during 14 consecutive days at dose of 100 mg kg^−1^ body weight.

### 4.3. Animals and Experimental Design

Fifty Y59 female growing rats, three months old, weighing 225 to 250 g, were used in this research. Animals were obtained from own breeding lab within the Department of Animal Physiology, Faculty of Science, University of Zagreb and maintained under a 12/12-h light-dark cycle with free access to food (4 RF 21, Mucedola, Settimo Milanese, Italy) and water and standard housing conditions (room temperature around 25 °C and 60% humidity).

Forty rats were administered *ig* with retinoic acid suspension (80 mg kg^−1^) once daily for 14 days. After 14 days, osteoporosis was successfully induced with retinoic acid suspension in the rat model as confirmed by bone mineral density (BMD). The values of BMD in randomly selected rats, in the proximal and distal metaphysis of the femoral neck were significantly lower in 13cRA group than in the control group (BMD-prox 0.242 ± 0.005 vs. 0.279 ± 0.013; BMD-dist 0.240 ± 0.002 vs. 0.282 ± 0.012). Subsequently, these rats were randomly allocated to one of four regimens for another 14 days: water (as the osteoporosis model control), alendronate of (40 mg kg^−1^) [1,29], proanthocyanidin (100 mg kg^−1^) [51] or icariin (100 mg kg^−1^). Additionally, a total of 10 healthy rats were treated *ig* with saline as the healthy control.

After 4 weeks all rats were anesthetized using a mixture of ketamine (Narketan^®^10, Vetoquinol AG, Belp Bern, Switzerland) at dose of 75 mg/kg with xylazine (Xylapana^®^ Vetoquinol Biowet Sp., z.o.o., Gorzów Wielkopolski, Poland) at dose of 10 mg/kg and their blood, left and right femurs, liver, kidney and uterine tissues were used for analyses. In this experiment the following parameters were analyzed: (1) body weight, blood and serum biochemistry for toxic effect of treatments; (2) femur weight ratio, bone histology, femur geometric characteristic, BMC, BMD, Ca/P and Vitamin D for their effect on bone health and metabolism; (3) Beta CrossLaps (β-CTx) and osteocalcin for their osteoclast and osteoblast activities; (4) antioxidant and anti-inflammatory markers for their anti-oxidation and anti-inflammation effects (5) uterine weight ratio for their estrogen effect.

The ethical committee (Faculty of Science, University of Zagreb, Croatia, No. 525-06-1-0255/10-5, 5 October 2010) approved present study and animal procedures were conducted according to the applicable laws and guidelines in the Republic of Croatia (Law on the Welfare of Animals, NN 102/2017, 18 October 2017), EU Directive 2010/63/EU and carried out in compliance with the Guide for the Care and Use of Laboratory Animals, DHHS Publ. # (NIH) 86-123.

### 4.4. Body Weight, Blood and Serum Biochemistry

During the experimental period, the body weight of each rat was monitored using a sensitive scale. The animals were weighed just before the start of the experiment (starting weight of animals 225–250 g) and during the experiment on days 6, 12, 18, 24 and 30 and body weight expressed in grams. The percentage change in weight of each animal was than calculated as follows: Percentage change in weight = Final weight-Initial weight × 100/Final weight. During the four-week dosing period, all the animals were monitored daily for clinical signs and any symptoms of toxicity, once before dosing, immediately after dosing and up to 1 h after dosing.

The blood samples were collected from the abdominal aorta in separator tubes and allowed to stand for 1 h to ensure complete clotting. Blood was centrifuged at 3000 rpm for 10 min and serum samples were used for the measurements of blood biochemistry, cytokines, vitamin D and bone turnover markers. Serum samples for measuring cytokines, Vitamin D and bone turnover markers were then frozen at −80 °C until biochemical assays were undertaken.

The following biochemical parameters from the blood were measured using the biochemical apparatus Becmann Coulter AU 680 (Beckman Coulter, Inc., Atlanta, GA, USA): aspartate aminotransferase (AST), alanine aminotransferase (ALT), alkaline phosphatase (ALP), urea, creatinine, blood glucose levels (glucose), lactate dehydrogenase (LDH), total protein and serum calcium (Ca) and phosphate (P) levels.

Blood for hematologic analysis was collected in vials containing potassium EDTA as anticoagulant and a fresh blood parameters was analysed by blood cell counter Cell-Dyn^®^ 3700 (Abbott, Lake Forest, IL, USA). Hematological parameters observed included number of erythrocytes (E), the average cellular volume of erythrocytes (MCV), haemoglobin (Hgb), haematocrit (Hct), mean cell haemoglobin (MCH), mean cell haemoglobin concentration (MCHC), total leukocyte count (L), and the total number of platelets (Plt).

### 4.5. Bone Analysis

The femurs were cleaned of adhering soft tissues and femur weight, length and the distal and proximal epiphyseal diameters of the femur were measured in medio-lateral (ML) and anterior-posterior directions (AP) by digital calliper as described in [20].

The left femur was used for analysis of bone mineral density (BMD) and area by dual-energy X-ray absorptiometry (DXA) on apparatus Hologic QDR^®^ 4000 DXA (Hologic Inc., Zaventem, Belgium). BMD were calculated by the bone mineral content (BMC) of the measured area and reported as g/cm^3^.

### 4.6. Bone Ash Contents, and Ca and P Analysis

For the Ca and P analysis, dissected right femurs were rinsed with deionized water, dried at 105 °C and then dry-ashed overnight in a muffle furnace at 450 °C (Gallenkamp). The ash residues were dissolved in concentrated nitric acid (HNO_3_, 65%, p.a. purity), heated and filled up after cooling to 10 mL with deionized water. Detailed analysis of calcium and phosphorus in ash is described in the paper [1]. Calcium was determined by atomic absorption spectrophotometry Varian SpectrAA-300 (Varian Inc., Palo Alto, CA, USA) at a wavelength of 422.7 nm. Phosphorus was determined *spectrophotometrically* at 660 nm (Cary 50; Varian Inc., Palo Alto, CA, USA).

Results are expressed as mg Ca or P g^−1^ of wet tissue weight. Accuracy of the methods was evaluated using Animal bone reference material H-5 (International Atomic Energy Agency, Vienna, Austria). The results of our analysis were within ± 10% of the reference values, i.e., 214.02 ± 1.81 mg g^−1^ for Ca (reference value: 212 mg g^−1^ wet weight) and 102.29 ± 0.31 mg g^−1^ for P (reference value: 102 mg g^−1^ wet weight).

### 4.7. Vitamin D Level and Biochemical Markers of Bone Turnover

Serum samples were used for the determination vitamin D and two parameters of bone turnover: osteocalcin (OC), a marker of bone formation and β-CrossLaps (β-CTx) marker of bone resorption. The **e**lectro**c**hemi**l**uminescence **i**mmuno**a**ssay “ECLIA” was used for the determination of markers using commercially kits for OC (N-MID Osteocalcin, Cobas-Roche diagnostic Ltd., Basel, Switzerland) and Beta CrossLaps (β-CTx, Cobas-Roche diagnostic Ltd., Basel, Switzerland). Total vitamin D (25-Hydroxy vitamin D) was analysed by the immunoassay analyser (Cobas-Roche, Basel, Switzerland). The measuring range for vitamin D, OC and CTXs is 3.00–70.0 ng/mL, 0.500–300 ng/mL and 10–6000 pg/mL, respectively.

### 4.8. Histological Examination of Rats’ Bone

The left femur was removed and fixed in 10% neutral buffered formaldehyde. After decalcification, each of the bones was cut at the mid shaft of the diaphysis, embedded in paraplast. Deparaplasted 6–7 μm thick cross sections of the femur distal diaphysis were stained with haematoxylin and eosin (HE) and examined under a light microscope (Nikon Eclipse E600, Vienna, Austria) at 40, 100 and 200× magnification. Photomicrographs were taken by digital camera (Nikon DMX1200, Vienna, Austria) and Imaging Software Lucia G 4.80 (Laboratory Imaging Ltd., Prague, Czechoslovakia).

### 4.9. Oxidative/Antioxidative Stress Markers Analysis in Liver and Kidney

Liver and kidney samples were prepared as described in paper [1]. The lipid peroxidation was determined by measuring the amounts of MDA via the thiobarbituric acid colour reaction at 532 nm and 600 nm with Libro S22 spectrophotometer (Biochrom Ltd., Cambridge, UK). Glutathione (GSH) assay and SOD activity were determined according to paper [52]. The measure of SOD activity is calculated from the percentage of inhibition of the reaction of xanthine oxidation by xanthine oxidase (optimized reaction ratio ΔA/min ≈ 0.025), which creates superoxide anion as a substrate for SOD present in samples. Catalase activity was determined by spectrophotometric method previously described [52].

### 4.10. Inflammatory Cytokines Analysis

Serum samples were used for Multi-Analyte ELISArray kit for rat (Kit: MER-004A of Quiagen and the levels of various cytokines (IL1α, IL1β, IL2, IL4, IL6, IL10, IL12, IL17A, IFNγ, TNFα, GM-CSF, and RANTES) were determined according to the manufacturer protocol as described in [52].

### 4.11. Uterine Weight

Uterus was dissected, washed with saline solution, and weighed on a digital analytical scale. Relative uterus index (mg/100 g) was calculated by dividing the uterus weight × 100 with the body weight.

### 4.12. Statistical Analyses

The data are expressed as mean ± SD. All data were analysed by Kruskal-Wallis ANOVA test. The differences between the different treatments were made with multiple comparisons of mean ranks for all groups. In all cases, a probability error of less than 0.05 was selected as the criterion for statistical significance. Statistical analyses were performed using STATISTICA 12 software (StatSoft, Tulsa, OK, USA).

## Figures and Tables

**Table 1:** Effect of icariin, proanthocyanidins and alendronate on enzymatic biochemical parameters in blood in rats with retinoic acid-induced bone loss.

Groups ^a^	Biochemical Parameters-Enzymes (X ± SD)					
	AST (U L^−1^)	ALT (U L^−1^)	ALP (U L^−1^)	GGT (U L^−1^)	LDH (U L^−1^)	Amylase (U L^−1^)
**Control**	78.33 ± 7.73	46.40 ± 9.52	170.13 ± 24.54	0.00 ± 0.00	464.50 ± 130.97	1751.00 ± 609.96
**13cRA**	96.16 ± 13.34	43.16 ± 4.66	224.00 ± 15.01 ^♦^	0.66 ± 0.51	725.66 ± 157.59	2046.16 ± 213.69
**Icariin**	83.00 ± 7.01	51.16 ± 5.19	164.00 ± 20.36	0.33 ± 0.51	385.83 ± 65.98 *	1549.66 ± 397.01
**Proanthoc**	85.16 ± 8.51	42.40 ± 6.94	186.66 ± 26.01	0.33 ± 0.51	286.50 ± 45.85 **	1831.66 ± 247.39
**Alendronate**	87.66 ± 7.50	42.00 ± 2.64	115.00 ± 12.00 **	0.33 ± 0.57	402.00 ± 122.74	2184.00 ± 419.06

^a^ Rats were administered *ig* with retinoic acid suspension (80 mg kg^−1^) once daily for 14 days. After inducing osteoporosis with 13cRA, rats were administered *ig* with icariin (100 mg kg^−1^), proanthocyanidins (100 mg kg^−1^) or alendronate (40 mg kg^−1^) once daily for next 14 days. Number of rats per group: 7. * Statistically significantly different compared to 13cRA (* *p* < 0.05; ** *p* < 0.01). ^♦^ Statistically significantly different compared to Control (^♦^ *p* < 0.05). Abbreviations: AST—aspartate aminotransferase; ALT—alanine aminotransferase; ALP—alkaline phosphatase; LDH—lactate dehydrogenase.

**Table 2:** Effect of icariin, proanthocyanidins and alendronate on leukocytes number and differential blood count in rats with retinoic acid-induced bone loss.

Groups ^a^	Leucocytes (10^9^ L^−1^)	Differential Blood Count (X ± SD)				
		Lymphocytes (%)	Monocytes (%)	Neutrophils (%)	Basophils (%)	Eosinophils (%)
**Control**	2.70 ± 1.17	79.06 ± 7.99	0.42 ± 0.21	13.46 ± 2.97	0.38 ± 0.21	0.61 ± 0.27
**13cRA**	3.88 ± 1.12	83.96 ± 2.74	0.45 ± 0.16	15.18 ± 2.28	0.55 ± 0.17	0.73 ± 0.36
**Icariin**	4.01 ± 1.01	78.01 ± 3.57	0.40 ± 0.18	20.15 ± 2.91	0.40 ± 0.16	0.70 ± 0.34
**Proanthoc**	4.70 ± 0.75	73.90 ± 4.81	0.35 ± 0.12	21.90 ± 3.13	0.66 ± 0.33	0.71 ± 0.24
**Alendronate**	5.35 ± 1.36 ^♦^	70.16 ± 3.29	0.86 ± 0.20 ^♦^	28.00 ± 4.33 ^♦^	1.23 ± 0.50	0.73 ± 0.41

^a^ Rats were administered *ig* with retinoic acid suspension (80 mg kg^−1^) once daily for 14 days. After inducing osteoporosis with 13cRA, rats were administered *ig* with icariin (100 mg kg^−1^), proanthocyanidins (100 mg kg^−1^) or alendronate (40 mg kg^−1^) once daily for next 14 days. Number of rats per group: 7. ^♦^ Statistically significantly different compared to control (^♦^ *p* < 0.05).

**Table 3:** Effect of icariin, proanthocyanidins and alendronate on relative bone weight in rats with retinoic acid-induced bone loss.

Groups ^a^	Relative Bone Weight (g/100 g)					
	Right Femur			Left Femur		
	X ± SD	Min Value	Max Value	X ± SD	Min Value	Max Value
**Control**	0.363 ± 0.014 **	0.350	0.386	0.347 ± 0.009 *	0.335	0.362
**13cRA**	0.314 ± 0.009	0.299	0.321	0.302 ± 0.012	0.285	0.316
**Icariin**	0.346 ± 0.004 *	0.341	0.355	0.333 ± 0.005	0.323	0.342
**Proanthoc**	0.333 ± 0.005	0.326	0.341	0.323 ± 0.017	0.303	0.342
**Alendronate**	0.373 ± 0.015 **	0.356	0.385	0.374 ± 0.036 *	0.351	0.416

^a^ Rats were administered *ig* with retinoic acid suspension (80 mg kg^−1^) once daily for 14 days. After inducing osteoporosis with 13cRA, rats were administered *ig* with icariin (100 mg kg^−1^), proanthocyanidins (100 mg kg^−1^) or alendronate (40 mg kg^−1^) once daily for next 14 days. The relative bone weight was expressed in g/100 g and was calculated as: Relative bone weight = Total bone weight × 100/Final body weight. Number of rats per group: 7. * Statistically significantly different compared to 13cRA (* *p* < 0.05; ** *p* < 0.01).

**Table 4:** Effect of icariin, proanthocyanidins and alendronate on femur geometric characteristics in rats with retinoic acid-induced bone loss.

Groups ^a^	Femur Geometric Characteristic (cm /100 g) (X ± SD)						
	AP φ Proximal Epiphysis	ML φ Proximal Epiphysis	AP φ Mid- Diaphysis	ML φ Mid- Diaphysis	AP φ Distal Epiphysis	ML Distal Epiphysis	Femur Length V. Trochanter-Condyle
**Control**	0.153 ± 0.004	0.223 ± 0.010	0.129 ± 0.004	0.172 ± 0.021 *	0.154 ± 0.009	0.207 ± 0.015	1.405 ± 0.050
**13cRA**	0.137 ± 0.009	0.214 ± 0.011	0.118 ± 0.005	0.141 ± 0.010	0.150 ± 0.008	0.213 ± 0.009	1.325 ± 0.044
**Icariin**	0.168 ± 0.010	0.247 ± 0.016	0.141 ± 0.006 **	0.149 ± 0.011	0.194 ± 0.010 **	0.236 ± 0.012	1.546 ± 0.043 ***
**Proanthoc.**	0.153 ± 0.008	0.236 ± 0.014	0.131 ± 0.007	0.145 ± 0.008	0.173 ± 0.014	0.235 ± 0.017	1.427 ± 0.096
**Alendronate**	0.146 ± 0.004	0.222 ± 0.005	0.128 ± 0.005	0.149 ±0.004	0.169 ± 0.015	0.228 ± 0.001	1.390 ± 0.036

^a^ Rats were administered *ig* with retinoic acid suspension (80 mg kg^−1^) once daily for 14 days. After inducing osteoporosis with 13cRA, rats were administered *ig* with icariin (100 mg kg^−1^), proanthocyanidins (100 mg kg^−1^) or alendronate (40 mg kg^−1^) once daily for next 14 days. The femur length was then measured with a digital sliding calliper from the top of the femur head to the distal point of the femur. The distal and proximal epiphyseal diameters of the femur were measured in medio-lateral (ML) and anterior-posterior directions (AP) by digital calliper. Number of rats per group: 7. * Statistically significantly different compared to 13cRA (* *p* < 0.05; ** *p* < 0.01; *** *p* < 0.001).

**Table 5:** Effect of icariin, proanthocyanidins or alendronate on content of calcium, phosphorus and vitamin D in femur and in serum in rats with retinoic acid-induced bone loss.

Groups ^a^	Content of Calcium and Phosphorus in Femur (X ± SD)		Content of Calcium and Phosphorus in Serum (X ± SD)		Serum 25-Hydroxy Vitamin D (nmol/L)
	Calcium (mg Ca/g)	Phosphorus (mg P/g)	Calcium (mmol/L)	Phosphorus (mmol/L)	
**Control**	134.50 ± 3.59 *	57.25 ± 1.25 *	2.31 ± 0.01	1.96 ± 0.15	28.1 ± 5.16
**13cRA**	121.00 ± 3.25	49.29 ± 2.15	2.39 ± 0.04	1.93 ± 0.26	20.60 ± 3.46
**Icariin**	138.80 ± 3.39 *	57.50 ± 1.94 *	2.32 ± 0.08	2.38 ± 0.18	25.08 ± 2.22
**Proanthoc.**	147.20 ± 2.15 *	58.80 ± 0.86 *	2.43 ± 0.11	2.32 ± 0.20	23.2 ± 2.11
**Alendronate**	136.00 ± 5.17 *	61.00 ± 2.41 *	2.46 ± 0.37	2.05 ± 0.15	22.71 ± 2.32

^a^ Rats were administered *ig* with retinoic acid suspension (80 mg kg^−1^) once daily for 14 days. After inducing osteoporosis with 13cRA, rats were administered *ig* with icariin (100 mg kg^−1^), proanthocyanidins (100 mg kg^−1^) or alendronate (40 mg kg^−1^) once daily for next 14 days. Number of rats per group: 7. * Statistically significantly different compared to 13cRA (* *p* < 0.05).

**Table 6:** Effect of icariin, proanthocyanidins and alendronate on kidney malondialdehyde (MDA) and glutathione (GSH) level and superoxide dismutase (SOD), and catalase (CAT) activities in rats with retinoic acid-induced bone loss.

Groups ^a^	Kidney (X ± SD)			
	MDA (nmol/mg of Kidney Proteins)	GSH (µg/mg of Kidney Proteins)	SOD (U/mg of Kidney Proteins)	CAT (U/mg of Kidney Proteins)
**Control**	4.09 ± 1.45 *	8.46 ± 0.22	4.07 ± 1.17	21.99 ± 3.85 *
**13cRA**	11.49 ± 3.09 ^♦^	3.60 ± 0.64 ^♦^	3.94 ± 1.28	8.93 ± 1.78 ^♦^
**Icariin**	5.75 ± 1.60	6.38 ± 2.06	3.51 ± 1.67	18.40 ± 4.27 *
**Proanthoc.**	4.63 ± 0.78 *	7.48 ± 2.11	3.04 ± 2.02	38.38 ± 5.94 *
**Alendronate**	9.84 ± 2.39	10.63 ± 1.05 **	7.74 ± 3.02 *	52.39 ± 6.92 **^◊^

^a^ Rats were administered *ig* with retinoic acid suspension (80 mg kg^−1^) once daily for 14 days. After inducing osteoporosis with 13cRA, rats were administered *ig* with icariin (100 mg kg^−1^), proanthocyanidins (100 mg kg^−1^) or alendronate (40 mg kg^−1^) once daily for next 14 days. Number of rats per group: 7. * Statistically significantly different compared to 13cRA (* *p* < 0.05; ** *p* < 0.01). ^♦^ Statistically significantly different compared to control (^♦^ *p* < 0.05). ^◊^ Statistically significantly different compared to icariin (^◊^ *p* < 0.05).

**Table 7:** Effect of icariin, proanthocyanidins or alendronate on liver malondialdehyde (MDA) and glutathione (GSH) level and superoxide dismutase (SOD), and catalase (CAT) activities in rats with retinoic acid-induced bone loss.

Groups ^a^	Liver (X±SD)			
	MDA (nmol/mg of Liver Proteins)	GSH (µg/mg of Liver Proteins)	SOD (U/mg of Liver Proteins)	CAT (U/mg of Liver Proteins)
**Control**	10.22 ± 1.37 *	8.89 ± 1.41	3.67 ± 1.68	4.98 ± 1.02
**13cRA**	14.05 ± 1.53 ^♦^	5.64 ± 1.09	1.66 ± 0.32	4.66 ± 1.49
**Icariin**	13.02 ± 1.89	9.83 ± 2.36 *	4.24 ± 1.03 *	4.67 ± 1.35
**Proanthoc.**	11.47 ± 2.15	5.96 ± 0.75	1.63 ± 0.16 ^◊^	4.32 ± 0.20
**Alendronate**	18.05 ± 5.17 ^♦^	5.08 ± 2.08	1.76 ± 0.35	4.07 ± 0.15

^a^ Rats were administered *ig* with retinoic acid suspension (80 mg kg^−1^) once daily for 14 days. After inducing osteoporosis with 13cRA, rats were administered *ig* with icariin (100 mg kg^−1^), proanthocyanidins (100 mg kg^−1^) or alendronate (40 mg kg^−1^) once daily for next 14 days. Number of rats per group: 7. * Statistically significantly different compared to 13cRA (* *p* < 0.05). ^♦^ Statistically significantly different compared to control (^♦^ *p* < 0.05). ^◊^ Statistically significantly different compared to icariin (^◊^ *p* < 0.05).

**Table 8:** Effect of icariin, proanthocyanidins and alendronate on relative uterine weight and relative bone weight in rats with retinoic acid-induced bone loss.

Groups ^a^	Phytoestrogenic Activity-Relative Weight of the Uterus (X ± SD)			
	Uterine Weight (g)	Increase (%) in Relation to 13cRA	Min. Value	Max. Value
**Control**	0.239 ± 0.029	19.49	0.205	0.263
**13cRA**	0.200 ± 0.011	-	0.182	0.216
**Icariin**	0.287 ± 0.035 **	43.42	0.237	0.336
**Proanthoc**	0.241 ± 0.031	20.91	0.213	0.283
**Alendronate**	0.235 ± 0.018	17.43	0.223	0.250

^a^ Rats were administered *ig* with retinoic acid suspension (80 mg kg^−1^) once daily for 14 days. After inducing osteoporosis with 13cRA, rats were administered *ig* with icariin (100 mg kg^−1^), proanthocyanidins (100 mg kg^−1^) or alendronate (40 mg kg^−1^) once daily for next 14 days. The relative uterine weight was expressed in g/100 g and was calculated as: Relative uterine weight = Total uterine weight × 100/Final body weight. Number of rats per group: 7. * Statistically significantly different compared to 13cRA (** *p* < 0.01).

## Abbreviations

BMD	bone mineral density
BMC	bone mineral content
VDR	vitamin D receptor
WHI	Women’s Health Initiative
MWS	Million Women Study
13cRA	13 cis-retinoic acid
*HRT*	*hormone replacement therapy*
OS	oxidative stress
SOD	superoxide dismutase
CAT	catalase
OC	osteocalcin
CTx	C-terminal telopeptide
β-CTx	β-CrossLaps marker of bone resorption
HE	hematoxylin and eosin
MDA	malondialdehyde
SDS	sodium dodecyl sulphate
DTNB	5,5′-Dithiobis(2-nitrobenzoic acid)
RDO	rapid decalcified
GSH	glutathione
AP	anterior-posterior length
ML	medio-lateral length
ROS	reactive oxygen species
